# 3D printing combined with anteroposterior cannulated screws for the treatment of posterolateral tibial plateau fracture

**DOI:** 10.1186/s12891-023-06887-9

**Published:** 2023-10-06

**Authors:** Zhihao Shen, Yingying Zhang, Feng Wu, Hua Chen, Huaizhi Ge

**Affiliations:** 1 Department of Orthopaedics Surgery, The Second Affiliated Hospital and Yuying Children’s Hospital of Wenzhou Medical University, NO.109, XueYuan West Road, Luheng District, Wenzhou, 325000 Zhejiang Province P.R. China; 2 Department of Radiology, The Second Affiliated Hospital and Yuying Children’s Hospital of Wenzhou Medical University, NO.109, XueYuan West Road, Luheng District, Wenzhou, 325000 Zhejiang Province P.R. China

**Keywords:** 3D printing, Posterolateral tibial plateau fracture, Orthopedics, Surgery

## Abstract

**Purpose:**

This study aimed to compare the effects of conventional surgery and three-dimension (3D) printing technology-assisted surgery in the treatment of posterolateral tibial plateau fractures (PTPF).

**Methods:**

A cohort of 61 patients afflicted with PTPF, spanning from June 2015 to October 2021, was enrolled. They were divided randomly into two groups: 31 cases of 3D printing group, 30 cases of conventional group. The personalized 3D-printed models were used to simulate the surgical procedures in 3D printing group. The demographic characteristics and clinical data were recorded, encompassing operation duration, intraoperative blood loss, intraoperative fluoroscopy shoots and fracture union time. The radiographic outcomes were gauged, encompassing tibiofemoral angle (FTA), tibial plateau angle (TPA), posterolateral slope angle (PSA) and Rasmussen’s anatomical score. The functional outcomes were assessed at the 12-month postoperative juncture, encompassing range of motion, Hospital for Special Surgery (HSS) score and Rasmussen’s functional score. Furthermore, fracture complications were evaluated,, encompassing infections, traumatic osteoarthritis, and delayed union.

**Results:**

The 3D printing group exhibited the operation time of 95.8 ± 30.2 min, intraoperative blood loss of 101.1 ± 55.3 ml, and intraoperative fluoroscopy shoots of 6.3 ± 2.3 times, while the conventional group recorded respective values of 115.5 ± 34.0 min, 137.0 ± 49.2 ml and 9.13 ± 2.5 times. Noteworthy disparities were evident between the conventional and 3D printing groups (p < 0.05). Furthermore, in comparison to the conventional group, the 3D printing group exhibited commendable radiological and functional outcomes both immediately and 12 months post-surgery, although statistical significance was not attained. Moreover, the 3D printing group experienced a paucity of complications compared to the conventional group, although without achieving statistical significance.

**Conclusion:**

This study demonstrated the clinical feasibility of 3D printing combined with anteroposterior cannulated screws for the treatment of posterolateral tibial plateau fracture.

## Introduction

Posterolateral tibial plateau fracture (PTPF) refers to the cortical rupture involving the posterolateral column of the tibial plateau [1, 2]. PTPF account for about 15% of all tibial plateau fractures and about 44% of lateral tibial plateau and bicondylar fractures [3–5]. The common injury mechanism is that the knee joint is subjected to axial and valgus violence during flexion, which causes the impact of femoral condyle on tibial plateau, resulting in coronal splitting fracture of posterior tibial condyle [6]. When subjected to high-energy injury, the fracture line can involve other quadrants of the tibial plateau, accompanied by fractures of the fibular head and significant soft tissue damage. On CT images, it typically appears as a deep depression on the posterolateral articular surface of the tibia plateau, which can be depicted as a “deep pit” on 3D reconstruction [7]. The clinical outcome of PTPF depends on the quality of reduction, and poor reduction is prone to postoperative complications such as knee instability and traumatic arthritis [8, 9].

Currently, there remains controversy surrounding the optimal surgical approach and fixation methods for addressing PTPF. Common surgical approaches include the posterolateral approach, posterior midline approach, and modified anterolateral approach, all of which can cause damage to the stable structures around the knee joint, as well as carry the risk of damaging surrounding blood vessels and nerves [9]. Sassoon et al. [10] reported that the use of anterior lateral steel plates does not adequately protect the effective support of the posterolateral plateau articular surface, and there is a high risk of fixation failure with this approach. On the other hand, Yang et al. [11] utilized cannulated screws or steel plates for effective internal fixation under arthroscopic assistance. This method was able to effectively restore the radiographic parameters of the tibial plateau and knee joint function postoperatively. However, arthroscopic-assisted knee surgery [12] cannot be effectively performed in most primary hospitals [8]. Therefore, it is worth exploring a surgical approach that can safely and effectively treat PTPF.

Adequate preoperative evaluation and accurate intraoperative manipulation are prerequisites for the success of complex tibial plateau fracture surgery [13]. On the contrary, inexperienced surgeons, inaccurate diagnosis, and improper selection of internal fixation are risk factors for surgical failure. Traditionally, imaging techniques such as computed tomography (CT), magnetic resonance imaging (MRI), and three-dimensional (3D) virtual visualization have been used as key tools for diagnosis and preoperative planning. However, images may obscure accurate recognition of orientation and dimension. Therefore, the application of 3D printing technology to develop PTPF models with precise anatomical features and quantitative feedback is helpful to improve the surgeon’s understanding of fracture, formulate personalized surgical plans, and strengthen doctor-patient communication [14].

In this study, 3D printing technology was utilized to reconstruct the PTPF of patients. Personalized treatment plans were developed through preoperative simulation on the 3D model, and PTPF were fixed with anteroposterior cannulated screws. The safety, feasibility, and effectiveness of the application of 3D printing technology in PTPF surgery were also evaluated.

## Materials and methods

### Patients

A total of 61 patients, who met the predefined inclusion and exclusion criteria, were enrolled in the study between June 2015 and October 2021. The inclusion criteria entailed as follows: (1) at least 18 years of age; (2) fresh closed fractures (within two weeks of injury); (3) PTPF or posterolateral fragments in tibial plateau fractures with a collapse of articular surface that exceeded 2 mm, as confirmed by CT (4). a minimum of 12 months of postoperative follow-up. The exclusion criteria encompassed as follows: (1) pathological fractures; (2) accompanied by local or systemic infection; (3) combined with vascular or nerve damage or calf compartment syndrome; (4) a history of knee joint dysfunction or knee surgery; (5) comminuted fracture. Using a random number table method, the patients were randomly allocated to two groups: the 3D printing group (31 cases) and the conventional group (30 cases). Both groups were well-matched in terms of age, gender, cause of injury, Schatzker classification [15], three-column classification [1], and concomitant injuries (Table 1). The surgical procedures, imaging evaluations and physical examinations were performed by the same orthopedic surgical team, and all patients provided informed consent and underwent regular follow-up for a minimum of one year. This study was approved by the Ethics Committee of the Second Affiliated Hospital of Wenzhou Medical University.

**Table 1:** Comparison of general characteristics between the two groups

	Conventional group (n = 30)	3D printing group (n = 31)	*p* value
Age, years	50.9 ± 14.3	54.0 ± 13.0	0.378
Gender, male	15 (50.0%)	16 (51.6%)	0.900
Hypertension	7 (23.3%)	9 (29.0%)	0.613
Diabetes	4 (13.3%)	3 (9.7%)	0.654
Side, left	17 (56.7%)	19 (61.3%)	0.714
Cause of injury			
Falling	16 (53.3%)	17 (54.8%)	0.338
Vehicle accident	12 (40.0%)	14 (45.2%)	
Direct impact	2 (6.7%)	0	
Schatzker classification			
type II	10 (33.3%)	14 (45.2%)	0.639
type V	14 (46.7%)	12 (38.7%)	
type VI	6 (20.0%)	5 (16.1%)	
Three-column classification			
Posterolateral column	2 (6.7%)	1 (3.2%)	0.790
Two columns	12 (40.0%)	14 (45.2%)	
Three columns	16 (53.3%)	16 (51.6%)	
Concomitant injuries			
Fibular head fracture	8 (26.7%)	13 (41.9%)	0.210
Meniscus injury	8 (26.7%)	11 (35.5%)	0.457
Cruciate ligaments injury	7 (23.3%)	4 (12.9%)	0.289
Collateral ligaments injury	4 (13.3%)	1 (3.2%)	0.331
Preoperative time, days	4.2 ± 1.6	4.5 ± 1.7	0.612
Follow-up, months	17.8 ± 4.3	18.7 ± 4.8	0.440

Mean ± SD or n, %

### Printing the 3D model

The CT scans of the patients’ knee joints were acquired from the Star PACS system (INFINITT, Seoul, South Korea) at the Second Affiliated Hospital of Wenzhou Medical University and saved in DICOM format. Subsequently, these data were imported into Mimics software version 18.0 (Materialise, Leuven, Belgium) for 3D reconstruction. By adjusting a range of parameters such as image contrast, image grayscale window, and employing region growing techniques, the tibial plateau’s structure and fracture fragments could be clearly displayed. The 3D digital models were later imported in STL format into Cura software version 15.04, where further modifications were made to the size and position. Finally, the 3D digital models in Gcode format were transferred to the 3D printer (X-maker, Qidi Inc, Zhejiang, China) using polylactic acid as the printing material. The 3D printer was configured with a layer height of 0.2 mm, a trace width of 0.4 mm, a filling density of 15%, a filling pattern of zigzag, and a printing speed of 100 mm/s.

### Simulated surgery

The 3D printing model enables the structural features of the fracture to be easily observed, and the surgeon can practice simulated fracture reduction and fixation operations on the models. Based on the morphology of the posterolateral fracture fragment in the tibial plateau, a bone fenestration with a size of 1 × 1 cm^2^ was performed on the anterolateral side of the tibia. The collapsed articular surface required pry-poking reduction and fixation with Kirschner wire. After the reduction of the posterolateral articular surface, anteroposterior cannulated screws with the appropriate length, position, and orientation were implanted on the model. The appropriate locking plate was then selected according to tibial plateau fractures at other positions. Finally, X-ray fluoroscopy was conducted on the model to evaluate the fracture reduction and internal fixation.

### Surgical procedure

The surgical procedure described involves the use of combined spinal-epidural anesthesia for the patient, followed by the placement of a pneumatic tourniquet on the thigh to control bleeding. The supra-fibular-head approach is used for posterolateral fractures of the tibial plateau, with a skin incision made below Gerdy’s tubercle and extending backwards and upwards, crossing over the fibular head and ending above the articular space. Careful separation of subcutaneous tissue, muscle, and fascia is performed to expose the articular capsule, with attention paid to avoid damage to the lateral collateral ligament and common peroneal nerve. The lateral meniscus is suspended with sutures to expose the posterolateral tibial plateau, and a bone fenestration is made on the anterolateral side of the tibia. The collapsed articular surface is reduced by pry-poking through the bone fenestration and fixed with Kirschner wires temporarily. Guide wires are then inserted into the tibial plateau, and anteroposterior cannulated screws are implanted after fluoroscopy with a C-arm X-ray machine. For fractures involving the lateral tibial plateau, a locking plate and screws are implanted for fixation. For fractures involving the medial tibial plateau, an additional medial incision is made for reduction and internal fixation. The knee is then imaged with anteroposterior and lateral X-ray radiographs taken by a C-arm X-ray machine. Finally, the wound is rinsed with normal saline, sutured layer by layer, and a drainage tube is placed subcutaneously.

In the 3D printing group, the surgeon used the 3D printed model to simulate the surgical procedure and determine the appropriate plates and screws based on the pre-determined position and direction. In contrast, in the Conventional group, the surgeon relied solely on their experience and intraoperative measurements to select the plates and screws.

### Perioperative management

Upon admission to our trauma center, all enrolled patients underwent a thorough medical history, physical examination, and routine preoperative evaluation. Radiological assessments, consisting of anteroposterior and lateral X-ray of the knee, along with CT, were carried out. Intravenous antibiotic prophylaxis was administered thirty minutes prior to the surgery, and again within 48 h post-operation, to prevent wound infections. The condition of the knee joint, regarding fracture reduction and fixation, was evaluated by X-ray on the second day after surgery, with removal of the drainage tube on the same day. Standard rehabilitation exercises were prescribed post-operation, with patients being advised to perform isometric quadriceps contractions and turn over in bed on the first day after surgery. During the initial week, continuous passive motion was performed using a machine, with gradual increments to knee flexion up to 90°. Patients were allowed to engage in touchdown weight bearing with the aid of crutches at 4 weeks post-operation, partial weight bearing at 12 weeks post-operation, and full weight bearing thereafter.

### Follow-up and evaluation criteria

**Fig. 1:**
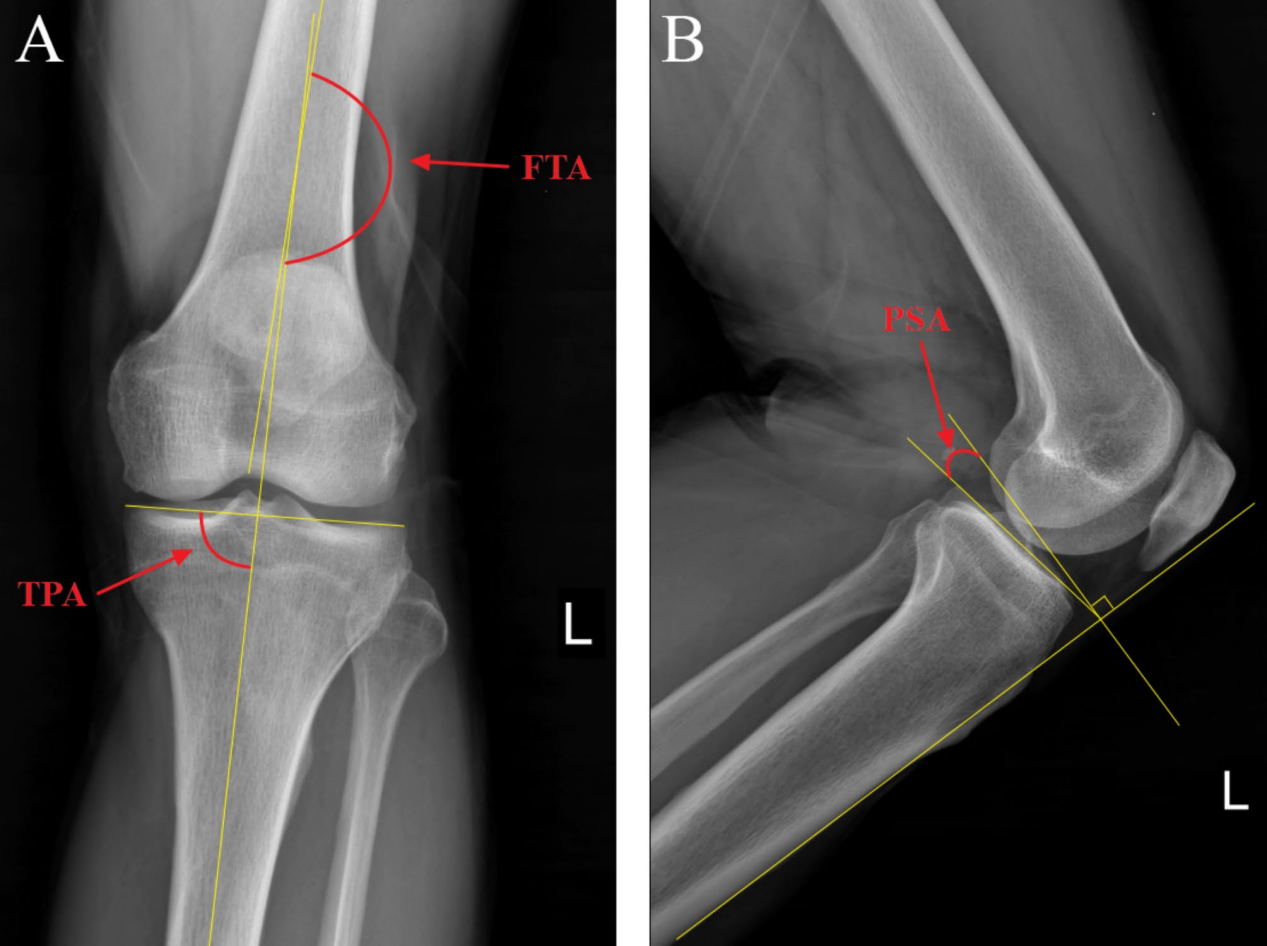
Schematic diagram of radiographic parameters of tibial plateau. **(A)** The FTA and TPA on anteroposterior X-ray. FTA is the lateral angle between the anatomical axes of the femur and tibia, TPA is the medial angle between the tangential line of the tibial plateau and the anatomical axis of the tibia. **(B)** The PSA on lateral X-ray. PSA is the lateral tibial plateau line and the perpendicular line of the anterior tibial cortex

Every patient underwent reviews at one and three months following the surgery, followed by reviews every three months and subsequently, annually after the completion of fracture healing. These follow-up visits involved medical history interviews, physical examinations, and radiographs of the affected knee joint. Clinical data of patients, such as operation time, intraoperative blood loss, intraoperative fluoroscopy shoots, hospital stay, and fracture union time were collected. The follow-up time at the point of fracture healing was recorded as the fracture union time. Radiographs were taken immediately after surgery and at the 12 months after surgery, to measure and record the tibiofemoral angle (FTA), tibial plateau angle (TPA), and posterolateral slope angle (PSA) [16], as seen in Fig. 1. Mal-reduction was defined as intra-articular step-off exceeding 2 mm; TPA ≥ 95°; PSA ≥ 95° or ≤-5° [17]. The quality of fracture reduction was evaluated using Rasmussen’s anatomical score [18]. Additionally, the range of motion (ROM), Hospital for Special Surgery (HSS) score [19], and Rasmussen’s functional score [18] at 12 months follow-up were measured to evaluate knee function. Primary and secondary outcomes, including fracture union and fracture complications such as infections, traumatic osteoarthritis, delayed union, malunion, and nonunion were assessed based on standard clinical and radiological criteria.

### Statistical analysis

The test for normality indicated that the sample data adhere to a normal distribution. Then the data were analyzed using Student’s *t* unpaired test and the chi-squared test. A p-value less than 0.05 was considered statistically significant. The data were presented as mean ± standard deviation (SD). The statistical analyses were conducted using SPSS software version 19.0 (SPSS Inc, Chicago, USA).

## Results

### General characteristics

In total, 61 patients were included in the study, of which 31 were administered 3D printing combined with anteroposterior cannulated screws. Therefore, there were 30 patients in the conventional group and 31 patients in the 3D printing group. The conventional group comprised 15 males and 15 females, while the 3D printing group consisted of 16 males and 15 females. The mean age of the conventional group was 50.9 ± 14.3 years and that of the 3D printing group was 54.0 ± 13.0 years. The most common cause of injury in both groups was falling. In accordance with the Schatzker classification, the conventional group exhibited 10 type II fractures, 14 type V fractures, and 6 type VI fractures, while the 3D printing group had 14 cases of type II, 12 cases of type V, and 5 cases of type VI. The conventional group displayed 2 cases of posterolateral column fractures, 12 cases of two-column fractures, and 16 cases of three-column fractures according to the three-column classification. In contrast, the 3D printing group had 1 case of posterolateral column fracture, 14 cases of two-column fractures, and 16 cases of three-column fractures. As for concomitant injuries, 8 patients in the conventional group had fibular head fracture, 8 had meniscus injury, 7 had cruciate ligament injury, and 4 had collateral ligament injury, whereas 13 patients in the 3D printing group had fibular head fracture, 11 had meniscus injury, 4 had cruciate ligament injury, and 1 had collateral ligament injury. The preoperative time was 4.2 ± 1.6 days in the conventional group and 4.5 ± 1.7 days in the 3D printing group. All patients were followed up for a minimum of 12 months, and there was no significant difference in the follow-up duration between the conventional group (17.8 ± 4.3 months) and the 3D printing group (18.7 ± 4.8 months). In summary, there were no significant differences between the two groups in general characteristics such as age, gender, cause of injury, Schatzker classification, three-column classification, concomitant injuries, preoperative time, and follow-up (Table 1).

### Clinical data

The clinical data results are presented in Table 2. The operation time for the 3D printing group was significantly shorter than the conventional group, with 95.8 ± 30.2 min compared to 115.5 ± 34.0 min, respectively (p < 0.05). The 3D printing group also had significantly less intraoperative blood loss than the conventional group, with 101.1 ± 55.3 ml and 137.0 ± 49.2 ml, respectively (p < 0.01). Moreover, the 3D printing group had a significantly lower number of intraoperative fluoroscopy shots than the conventional group, with 6.3 ± 2.3 and 9.13 ± 2.5, respectively (p < 0.001). However, there was no significant difference in the length of total hospital stays between the conventional group (11.4 ± 4.4 days) and 3D printing group (11.1 ± 3.0 days). Fracture union time was also recorded in both groups, and there was no significant difference between the 3D printing group (14.8 ± 3.2 months) and conventional group (16.6 ± 5.1 months).

**Table 2:** Comparison of clinical data between the two groups

	Conventional group (n = 30)	3D printing group (n = 31)	*p* value
Operation time, minutes	115.5 ± 34.0	95.8 ± 30.2	0.020^*^
Intraoperative blood loss, ml	137.0 ± 49.2	101.1 ± 55.3	0.010^*^
Intraoperative fluoroscopy shoots	9.13 ± 2.5	6.3 ± 2.3	0.000^***^
Total hospital stays, days	11.4 ± 4.4	11.1 ± 3.0	0.726
Postoperative hospital stays, days	7.9 ± 3.8	7.7 ± 4.0	0.901
Fracture union time, months	16.6 ± 5.1	14.8 ± 3.2	0.117

* p < 0.05, ** p < 0.01, *** p < 0.001

### Radiographic outcomes

The radiographic results are summarized in Table 3. Postoperative X-ray imaging demonstrated that both groups exhibited a certain degree of recovery in FTA, TPA, and PSA. The conventional group showed FTA of 171.5 ± 3.3° immediately after surgery and 172.0 ± 3.5° at the 12-month follow-up. Similarly, the 3D printing group had FTA of 171.0 ± 3.2° immediately after surgery and 171.6 ± 3.4° at the 12-month follow-up. No significant difference in FTA was observed between the two groups, with p values of 0.522 and 0.620 respectively. Furthermore, the conventional group exhibited TPA of 87.9 ± 2.8° and 88.6 ± 2.6° immediately after surgery and at 12 months after surgery, respectively. The 3D printing group showed TPA of 88.5 ± 2.5° and 88.6 ± 2.2° at these respective time points. There was no significant difference in TPA between the two groups, with p-values of 0.373 and 0.953. Furthermore, there were no significant differences in PSA between the conventional group and the 3D printing group at postoperative immediate and 12-month follow-up. The conventional group had PSA of 10.3 ± 2.7° and 10.3 ± 2.4°, while the 3D printing group had PSA of 10.2 ± 2.5° and 10.4 ± 2.7°, respectively, with p-values of 0.798 and 0.894. Combining these results with the definition of mal-reduction mentioned above, we found one case of mal-reduction in the conventional group, while there were no cases of mal-reduction in the 3D printing group. Besides, according to the Rasmussen anatomical score, there were 22 cases rated as excellent and 8 cases rated as good in the conventional group, while in the 3D printing group, there were 25 excellent cases and 6 good cases. There was no significant difference in Rasmussen anatomical score between the two groups (p = 0.505).

**Table 3:** Comparison of radiographic outcomes between the two groups

	Conventional group (n = 30)	3D printing group (n = 31)	*p* value
FTA, °			
Immediately after surgery	171.5 ± 3.3	171.0 ± 3.2	0.522
12 months after surgery	172.0 ± 3.5	171.6 ± 3.4	0.620
TPA, °			
Immediately after surgery	87.9 ± 2.8	88.5 ± 2.5	0.373
12 months after surgery	88.6 ± 2.6	88.6 ± 2.2	0.953
PSA, °			
Immediately after surgery	10.3 ± 2.7	10.2 ± 2.5	0.798
12 months after surgery	10.3 ± 2.4	10.4 ± 2.7	0.894
Rasmussen’s anatomical score	17.5 ± 0.9	17.6 ± 0.8	0.505

### Functional outcomes

Both groups reported substantial long-term improvements in knee joint symptoms and function at 12 months postoperatively compared to the initial situation. As shown in Table 4, the knee flexion motion of the conventional group was 128.0 ± 14.9°, while that of the 3D printing group was 133.1 ± 12.8°. The knee extension motion of the conventional group was 2.7 ± 1.9°, and that of the 3D printing group was 2.7 ± 2.1°. There was no significant difference in the range of knee joint motion between the two groups (p > 0.05). Furthermore, the HSS score in the conventional group was 90.5 ± 3.6, while in the 3D printing group it was 91.0 ± 2.9, and there was no significant difference between the two groups (p = 0.555). In addition, the Rasmussen’s functional score in the conventional group was 27.1 ± 1.9, while in the 3D printing group it was 27.2 ± 1.8. There was no significant difference in Rasmussen’s functional score between the two groups (p = 0.792). In terms of the Rasmussen’s functional score, in the conventional group, there were 25 patients rated as excellent, 4 were rated as good, and 1 was rated as fair. While, in the 3D printing group, 24 patients were rated as excellent, 6 were rated as good, and 1 was rated as fair. The 3D printing group showed a slightly higher rate of excellent-to-good functional results compared to the conventional group (96.8% vs. 96.7%), however, there was no significant difference between the two groups (p = 0.817).

**Table 4:** Comparison of functional outcomes between the two groups

	Conventional group (n = 30)	3D printing group (n = 31)	*p* value
ROM, °			
Extension	2.7 ± 1.9	2.7 ± 2.1	0.912
Flexion	128.0 ± 14.9	133.1 ± 12.8	0.159
HSS score	90.5 ± 3.6	91.0 ± 2.9	0.555
Rasmussen’s functional score	27.1 ± 1.9	27.2 ± 1.8	0.792
Excellent	25 (83.3%)	24 (77.4%)	0.817
Good	4 (13.3%)	6 (19.4%)	
Fair	1 (3.3%)	1 (3.2%)	
Rate of excellent-to-good	96.7%	96.8%	

### Complications

No vascular injury, fracture displacement, plate breakage, screw loosening, malunion or nonunion were observed in either group. However, some other postoperative complications occurred in both groups, which are summarized in Table 5. The overall incidence of complications in the conventional group and 3D printing group were 16.7% and 9.7%, respectively, with no statistically significant difference (p = 0.473). One patient in the 3D printing group had a superficial infection, while two patients in the conventional group had superficial infections, which were successfully treated with antibiotics and wound care. One patient in the conventional group had a deep infection, which was controlled by repeated debridement and the implantation of antibiotic-loaded bone cement spacers, and the wound eventually healed. However, the patient developed residual knee joint stiffness and poor flexion-extension function. The 3D printing group and the conventional group each had one case with evidence of traumatic osteoarthritis. After conservative treatment, they reported relief of knee pain, but still had limited squatting activity. One patient in both groups had delayed union, and they both recovered within 9 months after receiving enhanced weight-bearing and functional exercise.

**Table 5:** Comparison of complications between the two groups

	Conventional group (n = 30)	3D printing group (n = 31)	*p* value
Superficial infection	2 (6.7%)	1 (3.2%)	0.977^a^
Deep infection	1 (3.3%)	0	0.492^b^
Traumatic osteoarthritis	1 (3.3%)	1 (3.2%)	1.000^b^
Delayed union	1 (3.3%)	1 (3.2%)	1.000^b^
Malunion	0	0	
Nonunion	0	0	
Total	5 (16.7%)	3 (9.7%)	0.473^b^

^a^*p* value for continuity correction

^b^*p* value for Fisher’s exact test

### Typical case

**Fig. 2:**
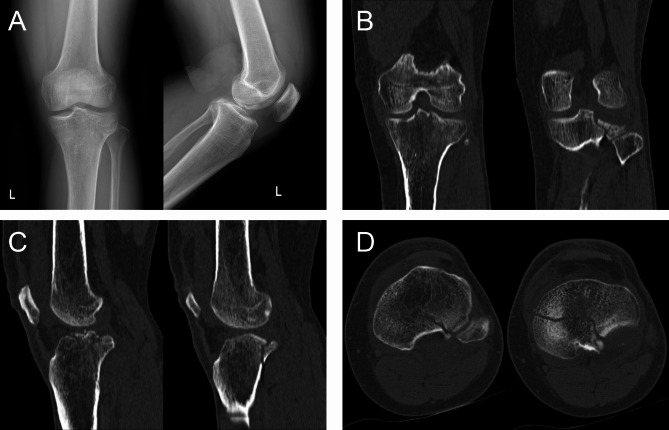
The patient’s preoperative radiological characteristics of PTPF. **A.** The anteroposterior and lateral X-ray. **B, C, D.** The coronal, sagittal and transverse CT images of PTPF

**Fig. 3:**
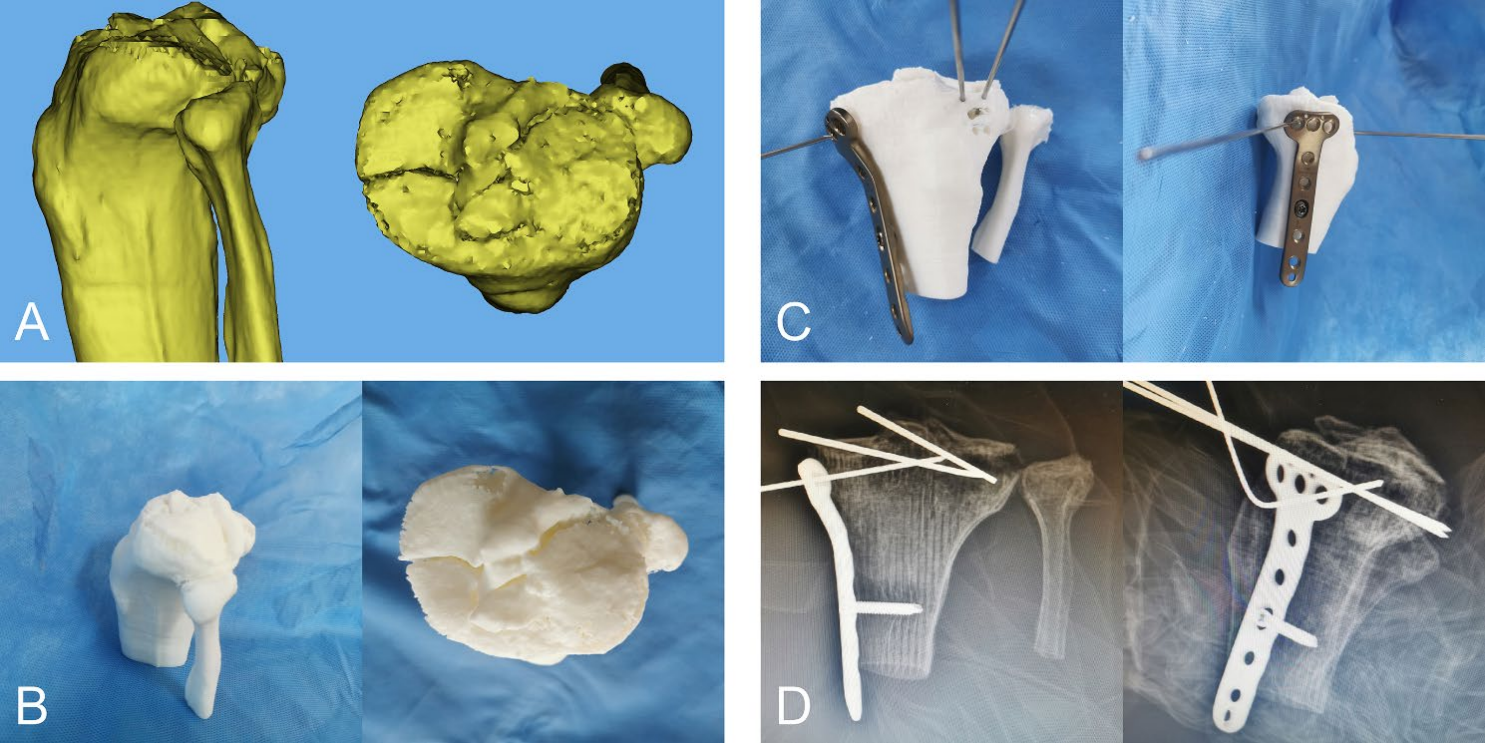
The 3D reconstruction and simulated surgery of the PTPF. **(A)** The 3D reconstruction of PTPF in Mimics software. **(B)** Preparation of simulative surgery. **(C)** Simulating the surgery on the 3D printed model. **(D)** The anteroposterior and lateral X-ray after the simulative surgery

**Fig. 4:**
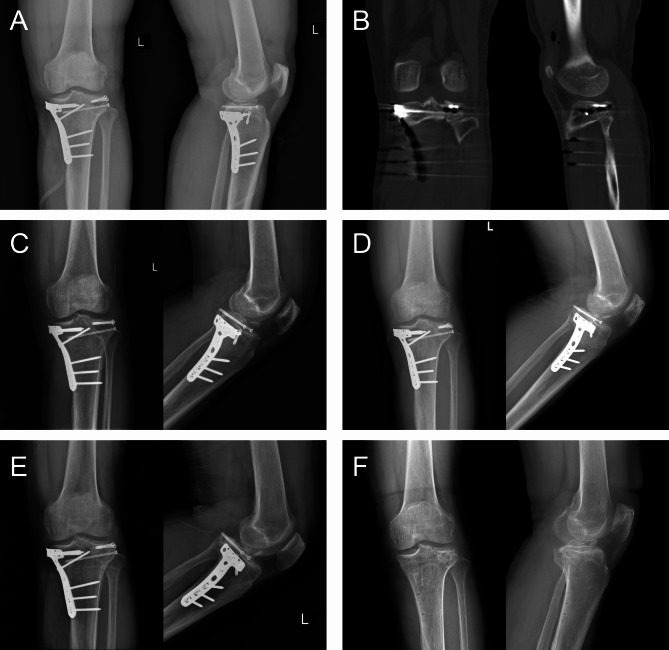
The patient’s radiographic images after the operation. **(A)** The anteroposterior and lateral X-ray after operation immediately. **(B)** The coronal and sagittal CT images after operation immediately. **C, D, E.** The anteroposterior and lateral X-ray at 1 month, 3 months and 12 months after operation. **F.** The anteroposterior and lateral X-ray at 15 months after operation (after internal fixation removal)

**Fig. 5:**
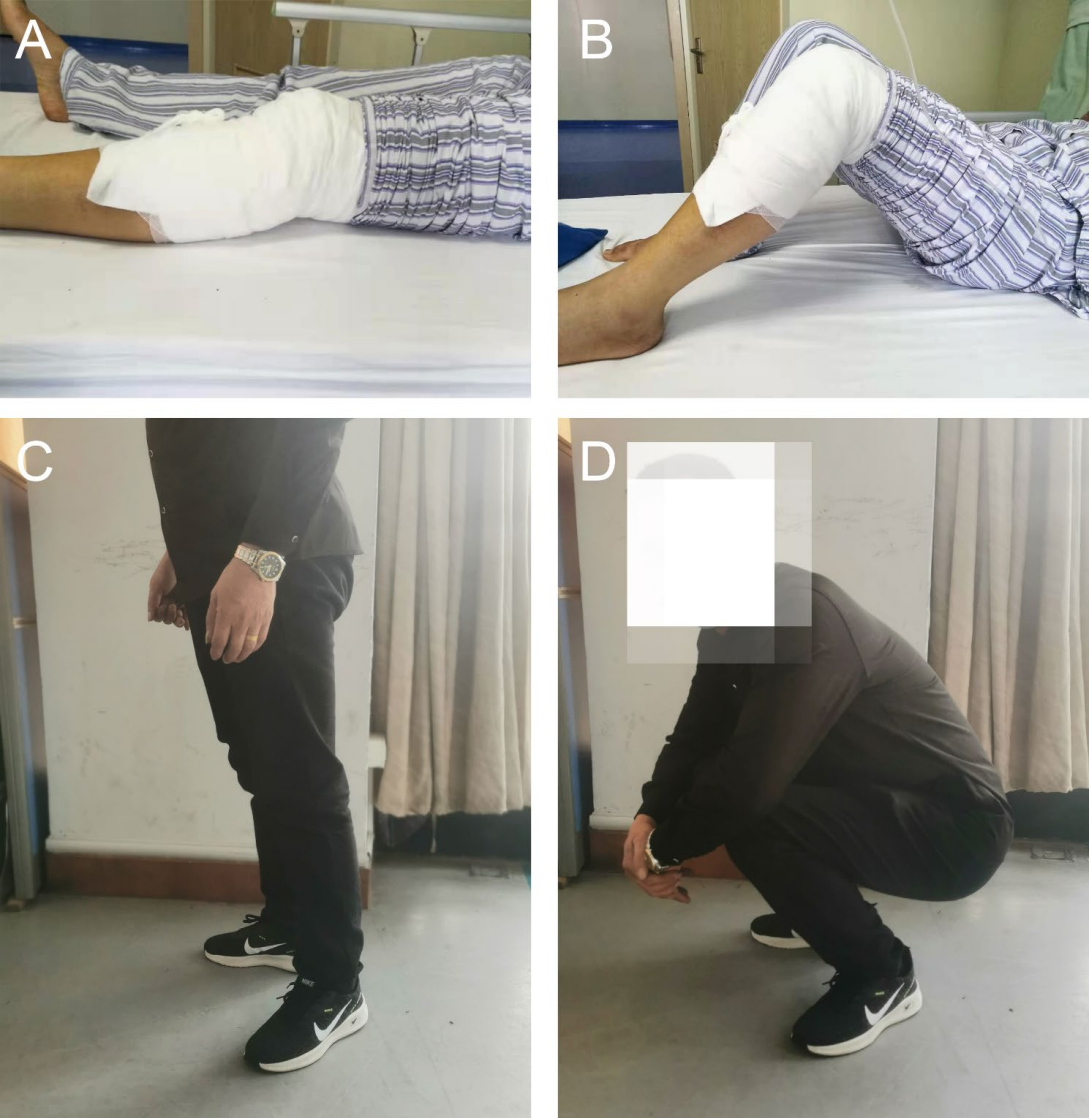
The patient’s postoperative range of knee join motion. **A, B.** The flexion and extension function of knee after operation immediately. **C, D.** The flexion and extension function of knee at 15 months after operation

Here, we present a case of a 49-year-old male who was admitted to our hospital due to pain, swelling and limited mobility of left knee joint for two hours after a traffic accident. Figure 2 shows the preoperative X-ray and CT scan of the fracture. The features of the injured tibial plateau were clearly displayed (Fig. 3) using the region growing and mask-editing technique of Mimics software version 18.0. Subsequently, an accurate 1:1 model of the injured tibial plateau was 3D printed, allowing the surgeon to observe and manipulate the exact replica of the fractured fragment for actual open reduction. The results of the simulated surgery are shown in Fig. 3. The actual surgery was then guided by the simulated surgery. Postoperative review of X-ray and CT images showed satisfactory fracture reduction and fixation (Fig. 4). The positioning of the plate and screws were good. The patient was followed up for 15 months during which he did not experience any surgical complications and demonstrated excellent Rasmussen’s functional score, as shown in Fig. 5.

## Discussion

PTPF remains a challenge for orthopedic surgeons, as it has only recently received attention in the field of orthopedics. The mechanism of violence in PTPF involves the knee joint being subjected to force while in a flexed position. Typically, this force exerts an axial stress or axial varus stress, resulting in an impact of the lateral femoral condyle on the posterior lateral aspect of the tibial plateau [20]. The collapse of this type of fracture occurs in the posterior part of the lateral tibial plateau, which is not easily detected in the anteroposterior and lateral X-ray images and is susceptible to misdiagnosis [7]. Moreover, this type of fracture often accompanies injuries to the fibula and soft tissues of the knee joint, hence the need for an MRI examination if necessary [21]. Therefore, a comprehensive preoperative understanding of the fracture’s anatomical structure is crucial for effective treatment.

The currently popular supra-fibular-head approach does not involve the exposure of the common peroneal nerve or popliteal vessels, nor does it require bone cutting or damage to other soft tissue structures [22]. However, this approach also has certain limitations, as it often requires a longer incision to achieve an adequate exposure. This study utilized a minimally invasive approach combining the supra-fibular-head approach with an anterior window, which allows for direct visualization of the posterolateral plateau and reduction of the fracture through cannulated lag screws from anterior to posterior. This method provides significant support for the collapsed articular surface, without damaging soft tissue or compromising knee stability, thus avoiding the risk of vascular or neural injury. The Rasmussen radiological scores of the 3D printing group showed no significant difference compared to those treated by traditional surgical methods, indicating that this minimally invasive approach is effective in reducing and maintaining the posterolateral plateau fracture.

In recent years, the use of 3D printing in surgical procedures has become increasingly widespread [23, 24]. Some studies have reported the advantages of 3D printing in the treatment of tibial plateau fractures. However, further exploration is needed for fractures that involve the posterolateral tibial plateau. These types of fractures are difficult to expose and fix, but with the use of 3D-printed physical models for preoperative simulation, the displacement of fracture fragments can be analyzed and effectively fixed. The specifications, quantity, and fixation position of implants can be determined, thereby reducing surgical time and intraoperative bleeding. Our study showed that the operation time for the 3D printing group was 95.8 ± 30.2 min, which was significantly shorter than the conventional group’s surgical time of 115.5 ± 34.0 min. Similarly, the intraoperative blood loss was also significantly lower in the 3D printing group (101.1 ± 55.3 ml) than in the conventional group (137.0 ± 49.2 ml). As the operation time and intraoperative blood loss decreased, patient recovery during the perioperative period was naturally accelerated. Although there was no statistical difference observed in the postoperative hospital stay between the 3D printing group and the conventional group (7.7 ± 4.0 days vs. 7.9 ± 3.8 days). Furthermore, these advantages have also been demonstrated in other bone fractures, particularly in complex fractures around the joints, such as pelvic fractures [25], pilon fractures [26], and elbow joint fractures [27]. With the aid of 3D printing models, one can gain a comprehensive understanding of the three-dimensional structure between fracture fragments, clarify the classification of fractures, and ascertain the relationships among the various fracture fragments.

In the widespread implementation of 3D printing technology for the treatment of PTPF, there still exist certain challenges. Primarily, it is paramount to acknowledge that the acquisition and upkeep of 3D printing technology entail preliminary costs, encompassing equipment, software, and personnel training. These initial investments necessitate equilibrium with potential long-term benefits, such as the reduction of surgical duration and associated expenses, as well as the diminishment of the requirement for various implant dimensions and the ensuing inventory costs. Furthermore, for surgical practitioners, the adoption of 3D technology necessitates not only a substantial commitment of time resources towards learning and preoperative strategizing, but also, to a certain extent, augments their occupational burden. Therefore, the cost-effectiveness of 3D printing in the healthcare domain necessitates a comprehensive assessment of multiple factors, including equipment investment, personnel training, personalized customization, and patient outcomes, among others. While the initial investments might be substantial, the potential for ameliorative effects and long-term savings renders 3D printing a promising investment in healthcare.

This study has some limitations and deficiencies. Firstly, the sample size of this study is insufficient, and there is a certain degree of systematic error. In the future, more prospective studies with larger sample sizes will be conducted to support the feasibility and unique advantages of this surgical approach. Secondly, the research team will delve deeper into the study of 3D printing, focusing on the research of 3D printed surgical guides and metal implants, truly achieving precision medicine. Thirdly, this study has not yet measured patients’ bone mineral density data. Given that variations in bone density might impact the healing process of fractures, the outcomes of this study could potentially harbor a degree of deviation.

## Conclusion

The application of 3D printing technology for preoperative simulation expedites operation time, reduces intraoperative blood loss, and minimizes the need for intraoperative fluoroscopy. The efficacy in terms of fracture reduction and functional recovery is comparable to conventional surgical methods. Therefore, the utilization of 3D printing combined with anteroposterior cannulated screws for treating posterolateral tibial plateau fractures holds practical clinical value.

## Data Availability

The datasets used and/or analysed during the current study available from the corresponding author on reasonable request.
